# Supplementary material for: Bioactive Metabolite Survey of Actinobacteria Showing Plant Growth Promoting Traits to Develop Novel Biofertilizers
Source: Metabolites. 2023 Mar 2;13(3):374. doi: 10.3390/metabo13030374 (PMC10052678; doi:10.3390/metabo13030374)
Supplement: Supplementary file 1 [file metabolites-13-00374-s001.zip › metabolites-2230036-SI.pdf]

Bioactive metabolite survey of actinobacteria showing plant growth-promoting traits to develop novel biofertilizers

Teresa Faddetta[1], Giulia Polito[1], Loredana Abbate[2], Pasquale Alibrandi[3], Marcello Zerbo[2], Ciro Caldiero[4], Chiara Reina[5], Guglielmo Puccio[1,2], Edoardo Vaccaro[3], Maria Rosa Abenavoli[4], Vincenzo Cavalieri[1], Francesco Mercati[2], Antonio Palumbo Piccionello[1*], Giuseppe Gallo[1,6*]

[1] Università degli Studi di Palermo, Dipartimento STEBICEF, Viale delle Scienze, 90128 Palermo (PA), Italy
[2] Consiglio Nazionale delle Ricerche, Institute of Biosciences and Bioresources (IBBR), Corso Calatafimi 414, 90129, Palermo (PA), Italy
[3] Mugavero Teresa S.A.S., Corso Umberto e Margherita n. 1/B, 90018 Termini Imerese (PA), Italy
[4] Università Mediterranea di Reggio Calabria, Dipartimento AGRARIA, località Feo di Vito, 89122 Reggio Calabria (RC), Italy
[5] Istituto di Candiolo - Fondazione del Piemonte per l'Oncologia - IRCCS Strada Provinciale, 142 -KM 3.95 - 10060 Candiolo (TO), Italy
[6] National Biodiversity Future Center, Piazza Marina 61, 90133 Palermo (PA), Italy
***** Authors to whom correspondence should be addressed: APP, antonio.palumbopiccionello@unipa.it, tel +3909123897544; GG, giuseppe.gallo@unipa.it, tel. +3909123897212.

Supporting information

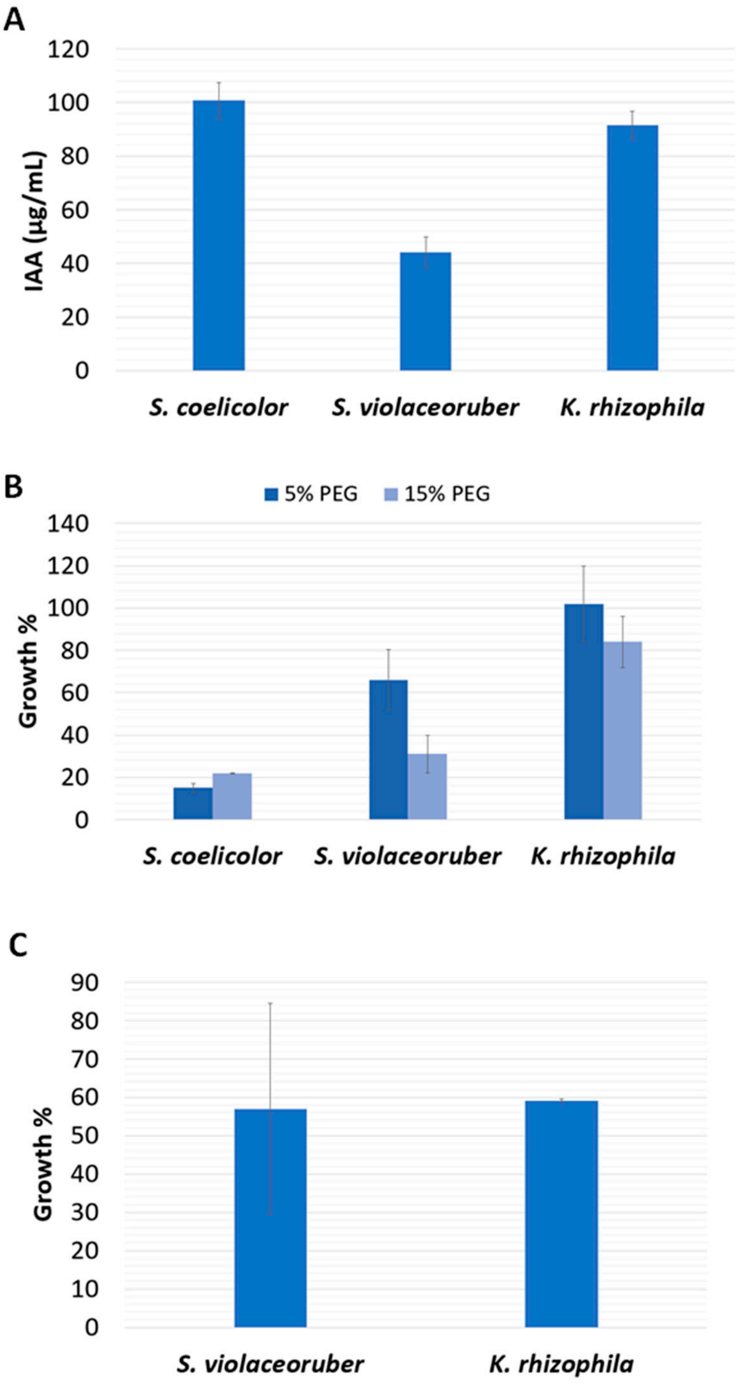

Figure S1 Characterization of PGP traits in the selected actinobacteria A) Indole-acetic acid (IAA) production by *S. coelicolor, S. violaceoruber* and *K. rhizophila* after 72 h of growth in R5A medium. B) Effect of drought stress on the growth of *S. coelicolor, S. violaceoruber* and *K. rhizophila* after 72 h of growth in R5A medium supplemented with 5 and 15% of PEG. C) Effect of salt stress on the growth of *S. violaceoruber* and *K. rhizophila* after 48 h of growth in R5A medium supplemented with 7.5% NaCl. In B and C, values are expressed as relative growth in respect to the same strains grown in the R5A without PEG or NaCl addition, respectively. The values are reported as mean of three cultivations; standard deviations are also reported.

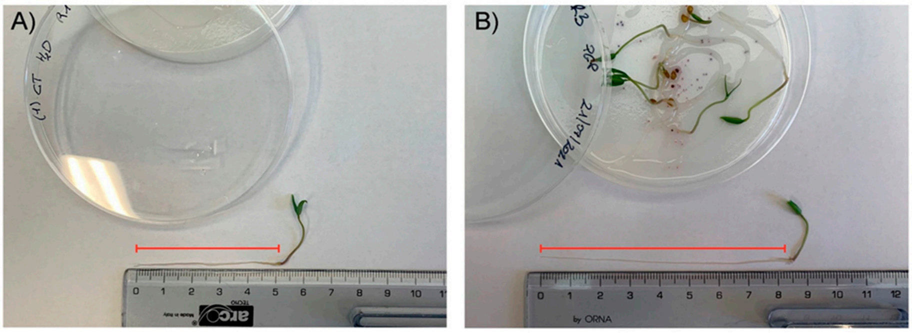

Figure S2. Effect of the selected PGP actinobacteria on seedlings regenerated from *S. lycopersicum* seeds on filter paper. As an example, panels A and B show a representative growth from untreated (control) and *S. vialoceoruber* treated seeds, respectively.
